# A Lagrangian Identification of the Main Sources of Moisture Affecting Northeastern Brazil during Its Pre-Rainy and Rainy Seasons

**DOI:** 10.1371/journal.pone.0011205

**Published:** 2010-06-18

**Authors:** Anita Drumond, Raquel Nieto, Ricardo Trigo, Tercio Ambrizzi, Everaldo Souza, Luis Gimeno

**Affiliations:** 1 EPhysLab, Facultad de Ciencias, Universidad de Vigo, Ourense, Spain; 2 University of Lisbon, CGUL-IDL, Lisbon, Portugal; 3 Universidade Lusófona, Departamento de Engenharias, Lisbon, Portugal; 4 Instituto de Astronomia, Geofísica e Ciências Atmosféricas, Departamento de Ciências Atmosféricas, Universidade de São Paulo, São Paulo, Brazil; 5 Universidade Federal do Pará, Belém, Brazil; University of Bristol, United Kingdom

## Abstract

This work examines the sources of moisture affecting the semi-arid Brazilian Northeast (NEB) during its pre-rainy and rainy season (JFMAM) through a Lagrangian diagnosis method. The FLEXPART model identifies the humidity contributions to the moisture budget over a region through the continuous computation of changes in the specific humidity along back or forward trajectories up to 10 days period. The numerical experiments were done for the period that spans between 2000 and 2004 and results were aggregated on a monthly basis. Results show that besides a minor local recycling component, the vast majority of moisture reaching NEB area is originated in the south Atlantic basin and that the nearby wet Amazon basin bears almost no impact. Moreover, although the maximum precipitation in the “Poligono das Secas” region (PS) occurs in March and the maximum precipitation associated with air parcels emanating from the South Atlantic towards PS is observed along January to March, the highest moisture contribution from this oceanic region occurs slightly later (April). A dynamical analysis suggests that the maximum precipitation observed in the PS sector does not coincide with the maximum moisture supply probably due to the combined effect of the Walker and Hadley cells in inhibiting the rising motions over the region in the months following April.

## Introduction

Values between 35% and 50% of the annual pluviometric total of the regional precipitation over the semi-arid northeastern region of Brazil (NEB) are observed during the austral autumn rainy season (March to May) [1]. Such rainy regime is mainly modulated by the seasonal migration of the Intertropical Convergence Zone (ITCZ) [2], which reaches its southernmost position in the equatorial south Atlantic around March-April [3], [4].

The interannual variability of precipitation of NEB constitutes currently one of the most appealing topics of study in tropical climatology. Although located well within the intertropical band, the northeastern region of Brazil receives considerably less annual precipitation than the neighboring Amazon region [5]. Water resources in the area suffer as a consequence of the combination of these unusual low values of annual precipitation (for a tropical region) with high values of interannual variability, influenced by several atmospheric and oceanic mechanisms, namely frontal systems, easterly waves, the ITCZ, and modulated by the El Nino and Atlantic signal [6]–[9]. Thus this densely populated area is relatively prone to drought episodes, with large socio-economic impact [10]. Nevertheless, despite the growing interest in the region and rising prospects for seasonal predictions [11], [12], it is not well known the exact location of moisture sources that affect NEB.

The characteristics of the precipitation depend, among other factors, on the available moisture. It is now commonly accepted that the precipitation that falls in a region has one of three origins [13]: (a) moisture already present in the atmosphere, (b) moisture advected into the region by wind, or (c) evaporation from the surface below. This last term corresponds to the recycling component. While definitions can vary, recycling is commonly defined as that part of the evaporated water from a given area that contributes to precipitation over the same area (for a review, see [14], [15]. Averaged over long periods, source (a) provides a negligible contribution. Therefore, two major processes are responsible for the observed atmospheric moisture: (i) local evaporation (recycling) and (ii) transport from remote sources (advection). Thus, it is extremely important to know the sources of the moisture that became precipitation in a given region. Trenberth [16] has already pointed out that the moisture contribution for the heavy and moderated precipitation does not result from local evaporation, but is associated with large distance transport and the low levels convergence are their main sources.

Stohl and James [17], [18] applied a Lagrangian method of diagnosis to determine the source of moisture in a basin. Their method is based on meteorological analysis data, a particle dispersion model, and a Lagrangian budget method for diagnosing the surface moisture flux. Using this methodology, Nieto et al. [19]–[21] identified the major sources of Sahel moisture, as well as of Iceland and Orinoco Basin, while Drumond et al. [22] investigated the main sources of moisture over Central Brazil and La Plata Basin, regions that coincide with two centers of action of a dipolar precipitation variability mode related to the South American Monsoon System (SAMS) [23].

This paper aims to identify the climatological main sources of moisture over NEB observed during the pre-rainy and the rainy seasons (from January to May) through the application of a Lagrangian methodology. Moreover we intend to analyze the seasonal variability of these sources and how these can be affected by the atmospheric circulation patterns at the regional scale.

## Materials and Methods

The Lagrangian method used is based on the calculation of a large number of trajectories with the particle dispersion model FLEXPART [17], [18], [24]. FLEXPART uses analysis data from the European Centre for Medium-Range Weather Forecasts [25] to calculate both the grid-scale advection as well as the turbulent and convective transport of so-called ‘particles’. There are various options for the generation of particles. In this case the atmosphere was “filled” homogeneously with particles, each representing a fraction of the total atmospheric mass. In other words, the atmosphere is divided into a large number *N* of so-called particles, which are homogeneously distributed such that their number density is proportional to the air density. Given a total atmospheric mass *ma*, each particle therefore represents a mass *m  =  ma*/*N.* A small error is introduced here because the mass of a particle (and, thus, also the mass of the whole atmosphere) is assumed to be constant. In reality, however, the atmosphere's mass changes slightly through the addition and removal of water. Then, these particles are transported by the model using 3D winds, with their positions and specific humidity (q) being recorded every 6 hours. The increases (e) and decreases (p) in moisture along the trajectory can be calculated through changes in (q) with time (e–p  =  m dq/dt), with (m) being the mass of the particle. When adding (e–p) for all the particles residing in the atmospheric column over an area we end up obtaining the aggregated (E–P), the surface freshwater flux, where (E) is the evaporation and (P) the precipitation rate per unit area. The method can also track (E–P) from a region backward in time along the trajectories, choosing particles appropriate for finding sources of moisture that can lead (but not necessarily) to precipitation. Full details describing the method and its limitations can be found in [17], [18].

In the work we used the tracks of 1,398,801 particles over a 5-year period (2000–2004) computed using ECMWF operational analysis available every six hours (00, 06, 12 and 18 UTC) with a 1°×1° resolution and all 60 vertical levels. (E–P) can be traced backwards or forwards from the region of interest every 6 hours, limiting the transport times to 10 days, which is the average time that water vapor resides in the atmosphere [26]. For the first trajectory time step, all the target particles resided over the defined regions and (*E–P*) is the region-integrated net surface freshwater flux. For subsequent trajectory time steps, (*E–P*) represents the net freshwater flux into the air mass travelling to (or from) each region in the case of backward integration (forward integration). The analysis of *(E–P)* values tells us where and when the moisture over both analyzed areas were received or lost. The calculations were done at the monthly time scale. (*E–P*) values for specific days can be labelled *(E–P)_n_* here, so *(E–P)_1_* shows where the moisture over the regions was received or lost on the first day of the trajectory. In order to better differentiate the results of both experiments in this work, the *(E–P)* averaged over days 1 to 10 is labelled *(E–P)^+10^* for a forward integration and *(E–P)^−10^* for a backward run. It is important to emphasize that the analysis of (E–P) values clarifies where and when the moisture of those particles reaching the atmosphere over the PS region was acquired or lost. We define the “moisture source region” as an area in which an air parcel either absorbed or lost significant amounts of moisture before reaching the atmosphere over the PS, and not just the sources of precipitation.

**Figure 1:**
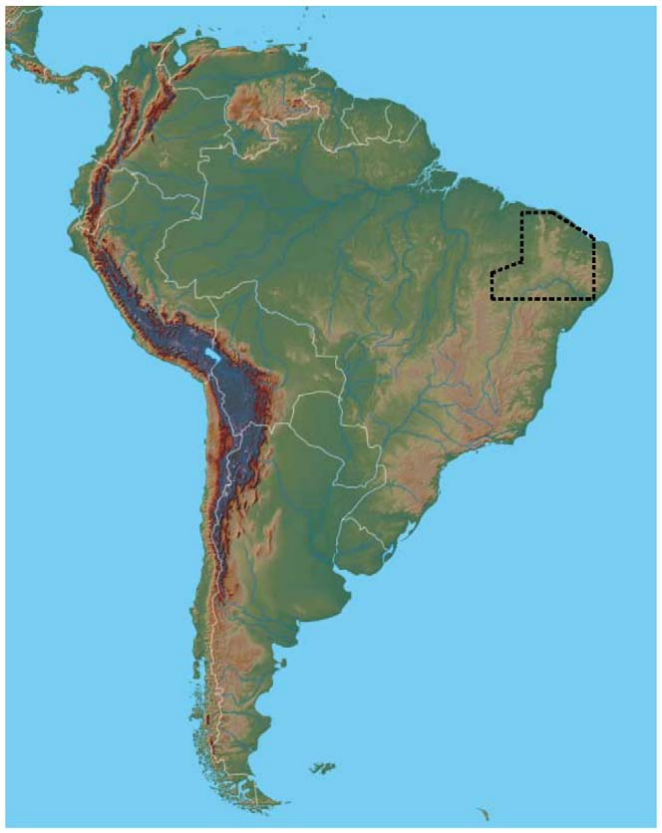
Box indicating the region of interest, so-called “Polígono das Secas” (PS).

Focusing on the semi-arid region, a box was designed isolating the so-called “Polígono das Secas” (PS), or drought-polygon (Figure 1). The spatial limits adopted for this domain are in accordance to those defined by the leading institution dealing with drought on the Brasilian “Nordeste” region - FUNCEME (http://www.funceme.br/DERAM/projetos/projeto_deram_bnb.htm) as well as the precipitation criteria found in Molion and Bernardo [27]. According to these works, this box encompasses a nearly homogeneous region which experiences a precipitation maximum from February to April, excluding the wetter coastal region of NEB.

A review of main climatological features associated with the configuration of NEB rainy season will be briefly presented together with some dynamical analysis intending to better understand the mechanisms that could be related to the temporal evolution of the moisture supply to the PS region. As the ECMWF analysis dataset used by the FLEXPART is not available for this kind of purpose, we will use the monthly means of vertical velocity and horizontal winds from the NCEP Reanalysis [28]. The sea surface temperature (SST) used was obtained from the NOAA Optimum Interpolation version 2 [29] while the precipitation data was retrieved from the Climate Prediction Center Merged Analysis Precipitation (CMAP) dataset [30]. The SST fields were extracted with the regular 1°×1° grid, while all the remaining datasets were gridded into the common global regular grid of 2.5°×2.5°. Monthly averages are analyzed over the 20°N – 20°S; 80°W – 0° sector.

For the dynamical analysis, the divergent wind components are obtained from the horizontal winds at each pressure level, following the same methodology as the one adopted by Souza et al. [1]. The divergent zonal (meridional) wind and the vertical velocity meridionally (zonally) averaged in the 10°S – 2.5°S (45°W – 35°W) band are displayed as vectors in the pressure-longitude (latitude) cross-sections, and these fields represent the divergent zonal (meridional) atmospheric circulations that describe the local Walker (Hadley) cell [31].

Before presenting the results, it is important to explain how to interpret the obtained patterns, which have been shown in recent works to provide a good representation of moisture source and sink regions affecting other tropical/subtropical areas [19]–[22]. Regions characterised by E–P>0 are represented by reddish colours while those characterised by E–P<0 are dominated by bluish ones. In the first case, evaporation dominates over precipitation, which indicates that air particles located within that vertical column (and bound to reach the analyzed areas in a backward case or that emanate from these regions in a forward integration) gain moisture. These regions are therefore identified as moisture source regions. In contrast, bluish colours (E–P<0) reveal regions where precipitation dominates over evaporation.

## Results

### 1. Monthly Climatology

**Figure 2:**
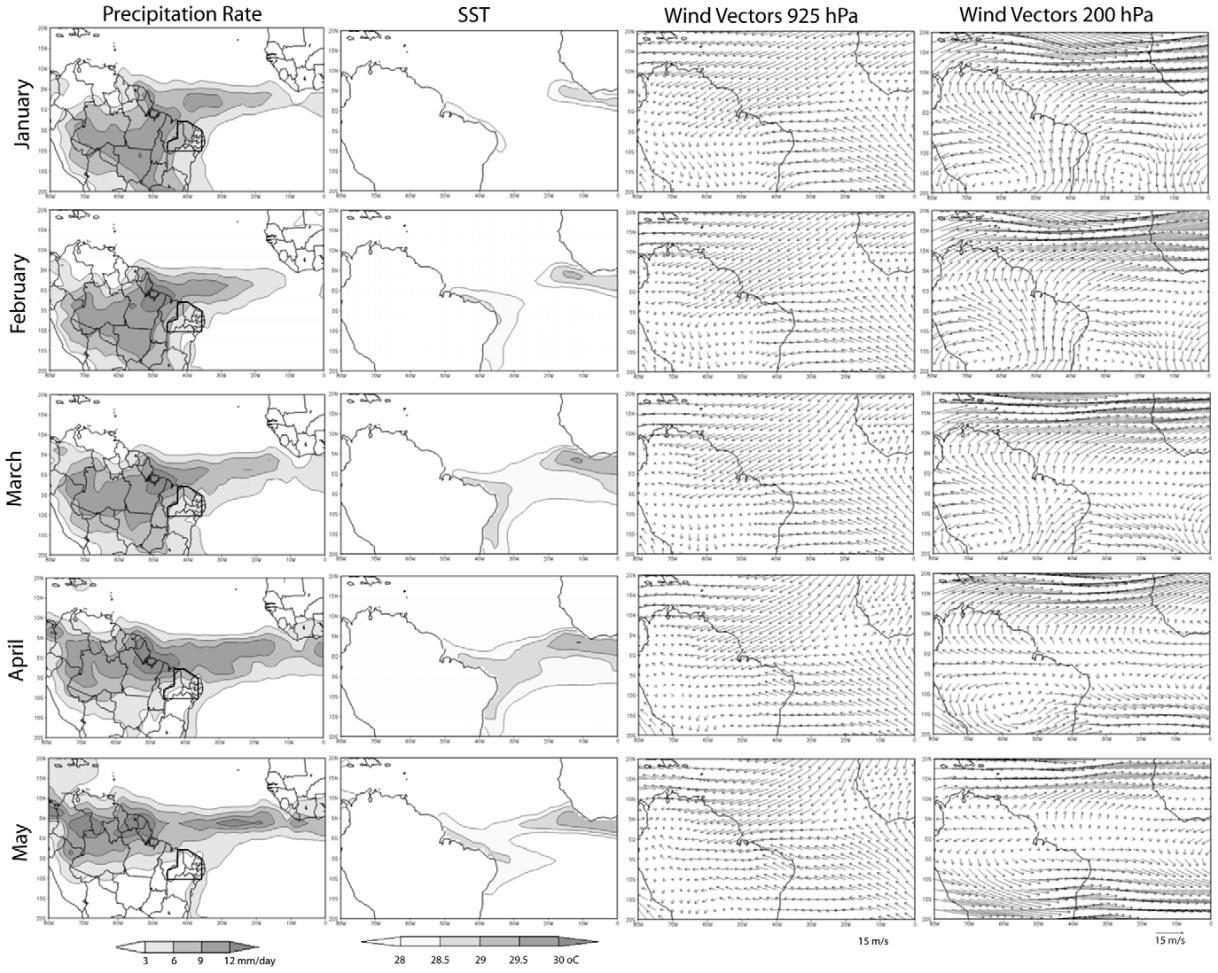
Precipitation, SST and wind monthly means from 2000 to 2004. Precipitation rate (mm/day) over near PS area (dotted line) (1^st^ column), SST (2^nd^ column), and 925 hPa and 200 hPa winds (3^rd^ and 4^th^ column respectively). For SST, only values greater than 28^o^C are plotted and shaded.

**Figure 3:**
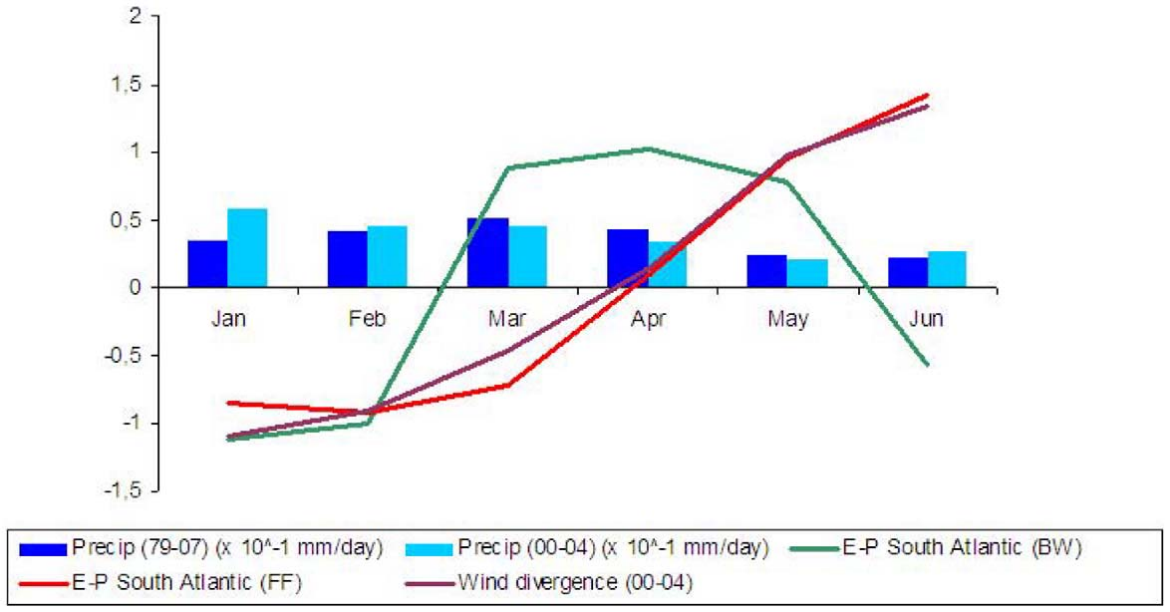
Precipitation, wind divergence and (E–P) time series calculated over PS region. i) PS precipitation rate mean (in mm/day divided by 10) for 1979–2007 (dark blue columns) and 2000–2004 (light blue columns); ii) 2000–2004 PS 850 hPa wind divergence (magenta line); iii) *(E–P)^−10^* over the PS area and integrated over the SA region (green line) and iv) *(E–P)^+10^* over the SA area and integrated over the PS region (red line). Except for precipitation, all series were normalized by its mean and standard deviation.

The monthly analysis showed here was elaborated using the 2000–2004 period to be coherent with the availability of the Lagrangian analysis results. Moreover, comparisons with previous analyses performed for longer periods help to confirm that this relatively short climatology period is quite similar to the long-term climatology. The evolution of monthly means of precipitation (figure 2, first column) show the migration of the precipitation during the pre-rainy and rainy season, with the highest values over the PS region being observed between January and March and a reduction in the subsequent months. In order to identify the seasonal cycle of precipitation for the region of interest, we have computed a spatial average for the PS delimited area. Obtained results confirm that the higher values occurred between January and March (figure 3). Note that in this figure the values of the precipitation time series are divided by 10 in order to have the same magnitude as the other curves presented. Although the ITCZ is in its southernmost oceanic position in March-April, the maximum precipitation over the studied area occurs slightly before, as it can be seen in the figures 2 and 3, suggesting that there are other mechanisms besides the ITCZ displacement that modulate the precipitation over the region, for instance, some influence of the SAMS. The pre-rainy and rainy season precipitation cycle obtained with the short (2000–2004) and long (1979–2007) confirms the resemblance of both climatological periods with the exception of the month of January. This could be related to some climatic variability mechanism that will not be discussed in this work, which focuses the precipitation cycle during the austral fall. Whatever, the T-test applied for the short (2000–2004) and long (1979–2007) January precipitation series indicates that there is no difference between the means.

In order to assess the most important underlying physical processes responsible for the seasonal evolution of precipitation we have also analysed the corresponding evolution of SST, lower (925 hPa) and upper (200 hPa) wind fields (Figure 2, second, third and fourth columns, respectively). The precipitation patterns obtained show the maximum extension of the oceanic ITCZ southwards in April and its migration northwards after that (figure 2, first column). There is a spatial expansion of the warmer waters over the Equatorial Atlantic during March, reaching the maximum values in April (figure 2, second column). Closer to NEB coast, one can also see the gradual expansion of warmer waters from the northern coast in February towards the rest of the region in the subsequent months.

The convergence of the low-levels winds (defined at 925 hPa) in the Equatorial Atlantic are also a good indicator of the ITCZ position (figure 2, third column). From the same sequence of plots it can be noted a wind convergence over NEB region, that also influences the precipitation cycle over the region. At higher levels (200 hPa), the winds (figure 2, forth column) are consistent with the precipitation patterns. As the maximum precipitation observed over central Brazil during January moves northwestward, the anticyclonic circulation located over central South America related to the Bolivian high and its associated downstream trough, moves equatorward and gets weaker along the analyzed period, until disappearing in May.

**Figure 4:**
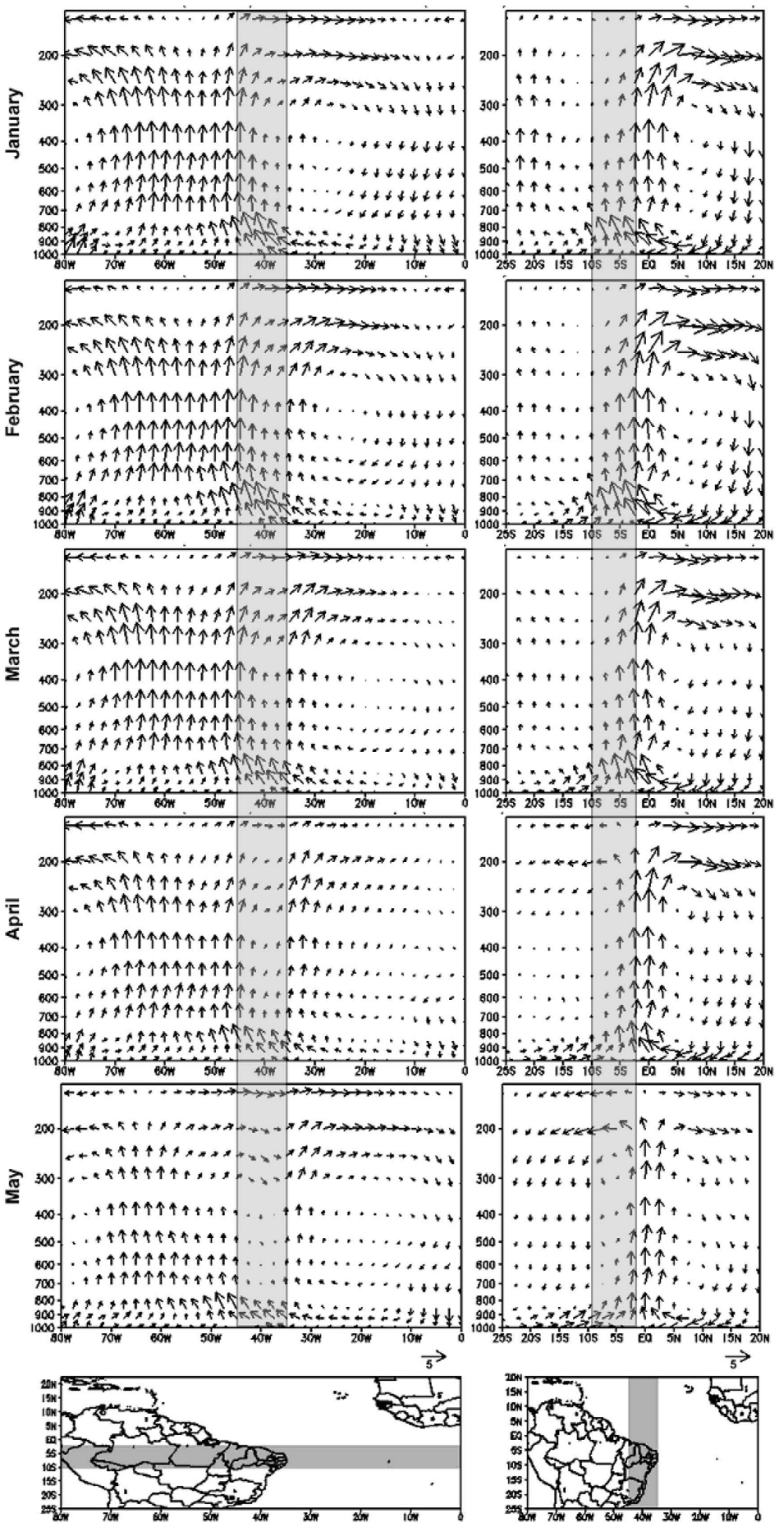
2000–2004 divergent atmospheric circulation vertical profiles. Monthly mean for the vectors composed by the divergent zonal wind component and the vertical velocity meridionally averaged in the *10^o^S – 2.5^o^S lat* band (left panel) and for the divergent meridional wind component and the vertical velocity latitudinally averaged in the *45^o^W – 35^o^W lon* band (right panel). The map at the bottom shows the selected bands (in grey), as well as the scale of the vectors (2 m/s; 2×10^−4^ hPa/s).

The evolution of the tropospheric circulation related to the regional branches of both the Walker and Hadley cells is shown in Figure 4. In general, strong ascending motion within troposphere over the Amazon longitudes is observed in the pressure-longitude cross-sections (fig. 4, left panels). This upward motion reaches upper levels where an outflow directed westward and eastward occurs and the upper-level eastward flow sinks over the Eastern Atlantic, where the easterlies prevail in the lower troposphere. As an outcome, it is possible to observe a cell configuration linking the Amazon to the Equatorial Atlantic. Analysing the temporal evolution (at the monthly scale), this ascending motion region is stronger and extends its domain towards NEB during January, February and March. In April this rising motion weakens and it almost disappears over NEB (45^o^W – 35^o^W). Finally, in May it is possible to observe some subsidence at higher levels over NEB and a new and weaker cell emerges over the Equatorial Atlantic. In the pressure-latitudes cross-sections, the rising motion over the equatorial region is accompanied by high levels divergence and by subsidence in the tropical northern hemisphere, in a structure similar to a cell (figure 4, right panels). At the monthly scale, there is a zonal displacement of the rising motion associated with the ITCZ migration, reaching its southernmost position during March and April. However, it is also possible to verify the higher intensity of the ascending movement between January and March, as well as the presence of a convergence over NEB (10^o^S – 2.5^o^S) at 750 hPa, with both these patterns weakening in the following months. We can note the occurrence of the ascending motions over the southern hemisphere subtropical latitudes during these three first months, being replaced by subsidence in May.

### 2. Air masses trajectories analysis

**Figure 5:**
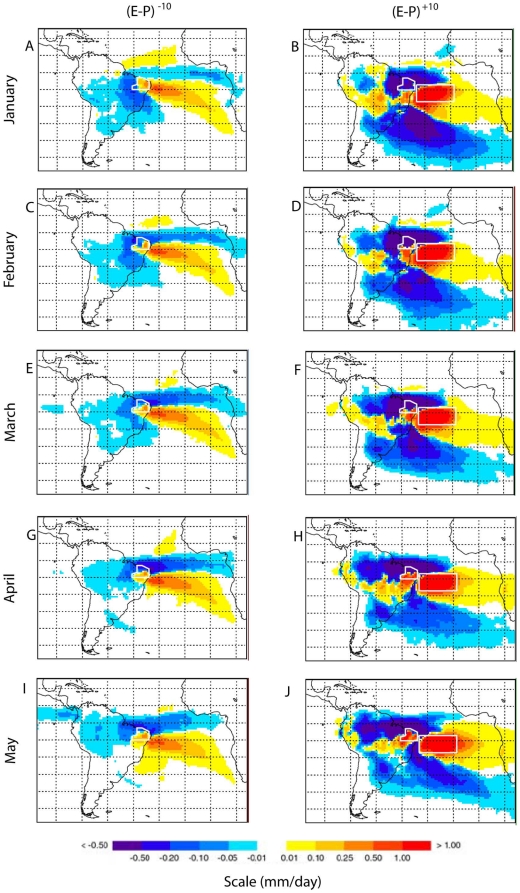
Monthly fields of (*E–P*) integrated along the 10 days for the period 2000–2004. Calculated backward tracking over the PS region (left panels, *(E–P)^−10^*) and calculated forward tracking over “South Tropical Atlantic” region (right panels, *(E–P)^+10^*).

In order to identify the origin of the air masses that reach the PS region during its pre-rainy and rainy season, all the particles residing in an atmospheric column over this area were tracked backwards in time to assess where they have gained or lost moisture. Monthly averages were computed over the previous 10 days period of transport (counting backwards). Through this backward trajectories analysis (figure 5, left column), one can confirm the major role played by the Tropical South Atlantic (TSA) in providing the bulk of moisture to the region (reddish colours). Although the Tropical North Atlantic (TNA) also appears as a moisture source, the air masses precedent from this area loose humidity when crossing the ITCZ region located on northern PS (bluish area). Local evaporation can be considered a minor contributing factor, while moisture advection from the South America (particularly from the wet Amazon basin) is negligible, as it can be seen through the predominance of moisture looses along the trajectories of the air masses coming from the continent (the bluish continental regions outside PS).

Quantifying the moisture contribution from TSA to PS, the *(E–P)_n_* series were calculated backwards from PS and integrated over the oceanic region delimited by 7°S–17°S; 35°W–13°W (see box in figure 5, right column). The results are showed in figure 3 and they indicate that the highest integrated contributions over 10 days occur between March and May, with the maximum taking place in April. It is important to mention that all temporal series presented in this figure were normalized (i.e. subtracting the JFMAMJ mean and dividing by the standard deviation related to the same period), with the exception of both precipitation time series, in order to facilitate the comparison between the time evolutions.

However, the air masses identified through the backwards trajectory might not be necessarily related with the precipitation in the region of interest because all particles (precipitating or not) are tracked back. Therefore, a forward analysis was also performed for this TSA box to find where the particles emanating from this oceanic area precipitate, which means looking for regions in the map that presented (E–P)^+10^ <0 (bluish colours) or the predominance of precipitation over evaporation (figure 5, right panels). These results suggest that the particles emanating from this box present higher looses of moisture over PS from January to March, decreasing in the following months. From the same figures one can see that the air masses emanating from TSA also loose moisture while crossing the ITCZ, Amazon and Subtropical Southern Atlantic regions (bluish areas), while they gain moisture travelling over the Western Central Brazil (reddish areas).

We have analysed the moisture contribution from TSA towards PS through an analysis where the *(E–P)_n_* series are computed forwards from the TSA and integrated over the PS region. From the figure 3 one can see that the higher integrated contributions over 10 days occur for the period between January and March, with the maximum taking place in February.

Summarizing, the maximum precipitation observed over PS occurs in March and the maximum PS precipitation originated from the TSA source occurs from January to March. However, the highest moisture contribution from this oceanic region to PS occurs slightly later (in April). Therefore, it seems that the period of highest looses of moisture presented by theses air parcels over PS does not coincide with the maximum moisture supply from TSA most probably due to some dynamical process that inhibits precipitation over the region.

Exploring the dynamics evolved, spatial means of the 850 hPa wind divergence over the PS region show a clear decreasing trend of the convergence from January onwards, with an inversion of the signal in April (figure 3). The tropospheric circulation evolution showed in figure 4 confirms this hypothesis indicating that there is a reduction of the rising motions over NEB during April as a consequence of the combined effect from the Walker and Hadley cells in inhibiting the rising motions over the region in the months following April.

## Discussion

An analysis of the sources of moisture over the semi-arid Brazilian Northeast (NEB) was performed using the FLEXPART Lagrangian method of diagnosis, focusing on the precipitation variability observed along its pre-rainy and rainy season (from January to May) and the relative importance of the Tropical South Atlantic in providing humidity during this period. The monthly mean conditions over a 5-year period (2000–2004) are studied.

The obtained results show that although the maximum precipitation in the “Poligono das Secas” region (PS) occurs in March and the maximum precipitation associated with air parcels emanating from the South Atlantic towards PS is observed between January and March, the highest moisture contribution from this oceanic region seems to happen with a temporal delay (in April). The dynamical analysis suggests that the maximum precipitation observed in PS during the 2000–2004 period does not coincide with the maximum moisture supply. This temporal mismatch may be related to the combined effect of the Walker and Hadley cells in inhibiting the rising motions over the region in the months following April.

Although the results discussed here are representative for a short time period (2000–2004), we believe they can contribute for a better understanding of the precipitation cycle over a region highly dependent of water in Brazil. We are confident that the application of this relatively new Lagrangian methodology (figure 5) and more standard compositing analysis (figures 2, 3 and 4) are particularly robust to accomplish this task. In any case, we are aware that a similar analysis using larger period of data is necessary to provide some information about the inter-annual climate variability over NEB. In order to answer this question, a more comprehensive analysis based on the FLEXPART model is currently being conducted using the 40 years long period of ECMWF Reanalyses and the results will be presented elsewhere.
